# Association between dietary choline and betaine intake and 10.6-year cardiovascular disease in adults

**DOI:** 10.1186/s12937-021-00755-9

**Published:** 2022-01-05

**Authors:** Mahdieh Golzarand, Parvin Mirmiran, Fereidoun Azizi

**Affiliations:** 1 Nutrition and Endocrine Research Center, Research Institute for Endocrine Sciences, Shahid Beheshti University of Medical Sciences, Tehran, Iran; 2 Department of Clinical Nutrition and Dietetics, Faculty of Nutrition Sciences and Food Technology, National Nutrition and Food Technology Research Institute, Shahid Beheshti University of Medical Sciences, No. 7, Shahid Hafezi St., Farahzadi Blvd., Shahrak-e-qods, Tehran, 1981619573 Iran; 3 Endocrine Research Center, Research Institute for Endocrine Sciences, Shahid Beheshti University of Medical Sciences, Tehran, Iran

**Keywords:** Choline, Betaine, Cardiovascular disease, Stroke, Mortality, Cohort

## Abstract

**Background:**

Several studies have assessed the association between dietary choline and betaine and cardiovascular disease (CVD), but their results are inconsistent. The present study aimed to determine the association between dietary intake of choline and betaine and the risk of CVD in the general population over a 10.6-year period of follow-up.

**Methods:**

The present cohort study was conducted on participants in the third wave of the Tehran Lipid and Glucose Study (2006–2008) and was followed-up until March 2018. Dietary intake of choline and betaine was calculated using the United States Department of Agriculture (USDA) database. Patients’ medical records were used to collect data on CVD.

**Results:**

In this study, 2606 subjects with no previous CVD participated and were followed-up for a median of 10.6 years. During the follow-up periods, 187 incidences of CVD were detected. Results of the Cox proportional hazards regression indicated that neither energy-adjusted total choline nor betaine was associated with the incidence of CVD. Among individual choline forms, only higher intake of free choline (FC) was associated with a lower risk of CVD (HR: 0.64, 95% CI: 0.42–0.98). There was no significant association between each 10 mg/d increase in choline and betaine content of each food category and CVD.

**Conclusion:**

Our investigation indicates no association between energy-adjusted total choline and betaine and a 10.6-year risk of CVD among adults. Besides, we found no relationship between individual choline forms (except FC) and CVD. We also found energy-adjusted choline and betaine obtained from food categories were not associated with the risk of CVD.

## Introduction

Cardiovascular disease (CVD) is a global health concern and is the leading cause of death in most countries. According to a large study, CVD affected 422.7 million people in the world in 2015 [1] and led to 17.9 million deaths (one-third of total mortality) every year [2]. CVD is comprised of coronary heart disease (CHD), cerebrovascular accident (CVA), peripheral artery disease (PAD), and atrial fibrillation; of those, ischemic heart disease (IHD) and stroke are the main causes of death worldwide [3], including Iran [4]. Some important risk factors for CVD are obesity, metabolic disorders, and an unhealthy diet, and improvement of these risk factors could reduce the risk of CVD [5].

Choline is an essential nutrient that is involved in one-carbon metabolism and the synthesis of neurotransmitters and cell membrane phospholipids [6]. Choline is synthesized in low amounts by humans; therefore, consuming choline from dietary sources such as eggs, meat, dairy products, whole grains, and vegetables is essential for providing adequate choline levels and maintaining optimal body functions [7, 8]. Recently, some studies have reported that a gut microbiota-related metabolite of choline, i.e., trimethylamine N-oxide (TMAO), is associated with an increased risk of CVD incidents and mortality [9–11]. Several studies have indicated an inverse association between dietary choline and betaine, a metabolite of choline, with some risk factors for CVD such as inflammatory markers [12] and hyperhomocysteinemia [13], but their results on the association with CVD are conflicting [14–16]. Besides, whether there is an association between choline and betaine and the risk of CVD is unclear [17]. Therefore, the present study aimed to determine the association between dietary intake of choline and betaine and the risk of CVD in the general population over a 10.6-year period of follow-up.

## Material and methods

**Fig. 1:**
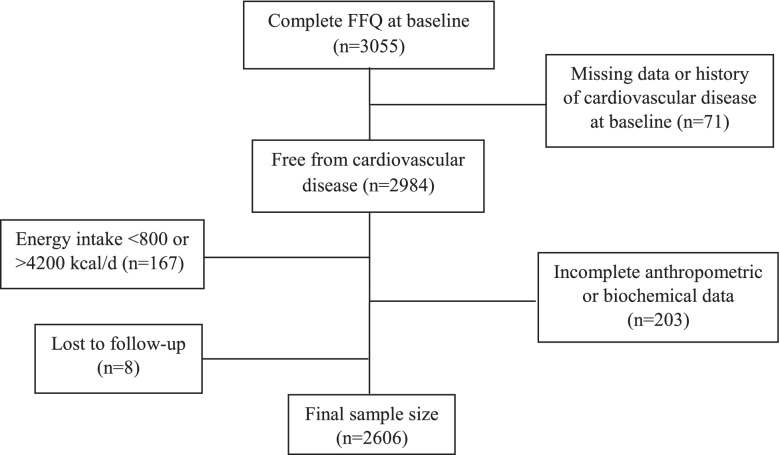
Flow chart of study

The present cohort study was conducted using data from subjects who had participated in the Tehran Lipid and Glucose Study (TLGS). The design and aims of TLGS have been reported previously [18]. In short, 15,005 males and females residing in district No. 13 of Tehran were enrolled and followed-up once every three years. For the current study, a total of 3055 subjects who had participated in the third wave of TLGS (2006–2008) and had complete dietary data were included. We excluded subjects with no data on CVD status, those who had a history of CVD events at baseline, or lost to follow-up (*n* = 79), total energy intake below 800 or above 4200 kcal/d (*n* = 167), and subjects with incomplete anthropometric or biochemical data (*n* = 203). Our final sample size included 2606 subjects that were followed-up until March 2018 (Fig. 1).

This study was conducted according to the Helsinki declaration of ethics. Written consent was obtained from all participants, and the ethical committee of Shahid Beheshti University of Medical Sciences approved the study.

### Assessment of covariates

Trained interviewers collected participants’ data, including age, sex, history of medications, and smoking status (yes vs. no), using a predefined questionnaire. According to the same protocol, the level of physical activity was estimated on the basis of a modifiable activity questionnaire (MAQ) [19] and anthropometric examinations, i.e., weight and height, were measured using a digital scale (Seca 707, Germany) and a tape measure, respectively. Then the body mass index (BMI) was calculated.

An enzymatic colorimetric assay was applied to detect fasting serum glucose (FSG) and lipid profile using the calibrated Selectra 2auto-analyzer device (Vital Scientific, Spankeren, Netherlands). This assay was based on glucose oxidase for measuring FSG, cholesterol esterase and cholesterol oxidase for total cholesterol, glycerol phosphate oxidase for triglycerides (TG), and phosphotungstic acid for high-density lipoprotein (HDL). All laboratory kits were provided by Pars Azmon Inc., Iran. Intra- and inter-coefficients of variation for glucose were 2.2%, for triglycerides they were 0.6 and 1.6%, and for HDL they were 0.5 and 2% [20].

Systolic blood pressure (SBP) and diastolic blood pressure (DBP) were determined following 15 min of rest in a seated position on the right arm using a mercury sphygmomanometer (Riester, Germany).

### The definition of high-risk patients

High-risk patients include patients with obesity, or type 2 diabetes, or dyslipidemia, or hypertension. Obesity was defined as a BMI ≥ 30 kg/m^2^ [21]. Diabetes was defined as having an FSG ≥ 126 mg/dL or using antidiabetic medications [22]. Dyslipidemia was determined based on the following criteria: TG ≥ 150 mg/dL, or HDL < 40 mg/dL, or low-density lipoprotein (LDL) ≥ 140 mg/dL, or taking antihyperlipidemic medications [23]. Hypertension was defined as SBP ≥140 mmHg, DBP ≥ 90 mmHg, or use of antihypertensive mediations [24].

### Assessment of dietary intake and dietary choline and betaine

The dietary intake of participants was collected using a validated 168-item food frequency questionnaire (FFQ) [25]. For this, the frequency (e.g. daily, weekly, and monthly) and portion size (e.g. cup, spoon, and ounce) of each consumed food item during the last 12 months were asked by trained interviewers at baseline. Then foods’ portion sizes were converted to grams to calculate participants’ total energy intake and grams of each food for estimating energy-adjusted dietary choline and betaine intake. In addition, food items were classified into larger categories, for example, grains, fruit, etc., and reported.

In the current study, the total amount of dietary choline and betaine from each food item was estimated using the United States Department of Agriculture (USDA) database for the choline content of common foods (Release Two, 2008) [26]. Accordingly, milligrams of total choline, individual choline forms (i.e., free choline (FC), glycerophosphocholine (GPC), phosphocholine (PC), phosphatidylcholine (PtC), sphingomyelin (SM)), and betaine per gram of each food were calculated. Then the total amount of choline and betaine for each food category was estimated. Among the 168 food items in our FFQ, data about choline and betaine for 104 food items was available in the USDA database and estimation was conducted based on available foods. Total Choline refers to the sum of FC, GPC, PC, PtC, and SM. Betaine is not included in the sum.

### Ascertainment of cardiovascular events

Information about cardiovascular events, mortality, and specific causes of mortality is updated annually by a trained nurse. Then a physician collected the patients’ medical records or death certificates for more evaluation. In the end, the outcome committee of TLGS assessed the outcome data and judged events. The CHD cases included definite and probable myocardial infraction (MI), unstable angina, angiographic verified CHD, heart failure, and CHD mortality. A stroke is sometimes characterized as a definite or prospective stroke or a transitory ischemic event. The CVD was defined as a CHD, stroke, or CVD-related mortlity [27]. All diagnoses were based on the 10^th^ edition of the International Classification of Diseases codes (ICD-10).

### Statistical analysis

In the present study, continuous variables were reported as geometric mean (95% prediction interval) and categorical variables as count (%). The residual model was applied to adjust total choline, individual choline forms, and betaine intake for the total energy intake [28]. Cox proportional hazards models were conducted to explore the relationship between total choline and betaine intake and the risk of CVD incidents. The hazard ratio (HR) and 95% CI for the incidence of CVD were reported as continuous (i.e., per 50 mg/d increase in choline and 25 mg/d increase in betaine) and categorized (i.e., per quartile). We also assessed the relationship between individual choline forms and choline and betaine content of food categories with CVD incidents. The person-years for each participant were calculated from baseline to the date of the first cardiovascular incidence reported, the date of death, or the end of follow-up. Some covariates, such as age, sex, BMI, total energy, and smoking, were selected a priori. The other covariates were selected using a data-driven method (change-in-estimate criterion) [29]. Accordingly, variables were included in the final model if they changed the ratio of total choline/betaine to CVD risk by > 5% in the final model [30]. The first model was adjusted for age and sex. For choline, the second model was adjusted based on age, sex, total energy intake, smoking, FSG, TG/HDL ratio, and dietary intake of meat, fruit, vegetables, and oil. For betaine, we adjusted the second model with age, sex, total energy intake, smoking, FSG, TG/HDL ratio, SBP, and dietary intake of fruit, total fiber, and oil. The proportional hazards (PH) assumptions in the Cox model were checked using the Schoenfeld residuals. The restricted cubic spline was performed to assess the nonlinear relationships between total choline and betaine and the risk of CVD by adjusting variables. We also conducted a sensitivity analysis to investigate the relationship between choline and betaine and CVD among healthy adults and high-risk patients. Data was analyzed using SPSS software (version 20.0; IBM Corporation, Armonk, NY, USA).

## Results

In the present cohort study, 2606 subjects without CVD history were participants (Fig. 1). The median follow-up period was 10.6 years. The mean baseline age was 37.1 years, and 45.1% of the participants were males. A total of 6.63% of participants suffered from diabetes, 25.0% from obesity, 53.1% from dyslipidemia, and 12.9% from hypertension. The general characteristics of the participants are presented in Table 1.

**Table 1:** General characteristics of participants (*n* = 2606)

Characteristics	Total cohort
Age (year)	37.1 (36.6–37.6)
Male (%)	1176 (45.1)
Body mass index (kg/m^2^)	26.5 (26.4–26.7)
Fasting serum glucose (mg/dL)	89.6 (89.0–90.2)
Total cholesterol (mg/dL)	182 (180–183)
Triglycerides (mg/dL)	122 (120–125)
High-density cholesterol (mg/dL)	41.7 (41.3–42.1)
Low-density cholesterol (mg/dL)	110 (109–112)
Systolic blood pressure (mmHg)	110 (109–111)
Diastolic blood pressure (mmHg)	72.6 (72.2–73.0)
Smoking (%)	242 (9.28)
Obesity (%)	652 (25.0)
Type 2 diabetes (%)	173 (6.63)
Dyslipidemia (%)	1385 (53.1)
Hypertension (%)	335 (12.9)
Physical activity (Met-min/wk)	1210 (1150–1275)

Data presented as geometric mean (95% prediction interval) for continuous and count (%) for non-continuous variables

The geometric mean dietary intake of energy-adjusted total choline was 226 mg/d and energy-adjusted betaine was 78.0 mg/d. The most important food sources of dietary total choline were meat (21.6%), grains and bakery products (19.7%), and dairy products (19.6%), and for betaine were grains and bakery products (72.2%), followed by fruit and vegetables (11.5%) (Table 2).

**Table 2:** Dietary intake of total choline, betaine, individual choline forms, and their intakes from each food category (n = 2606)

Dietary intake	Total cohort	Contribution (%)
Dietary energy (kcal/d)	2140 (2115–2166)	–
Energy-adjusted total choline (mg/d)	226 (223–228)	–
Energy-adjusted betaine (mg/d)	78.0 (76.7–79.3)	–
Free choline (mg/d)	56.9 (56.4–57.5)	26.1
Glycerophosphocholine (mg/d)	10.9 (10.8–11.1)	19.8
Phosphocholine (mg/d)	42.2 (41.6–42.9)	5.13
Phosphatidylcholine (mg/d)	94.6 (93.0–96.1)	43.5
Sphingomyelin (mg/d)	9.57 (9.40–9.75)	4.43
Choline-meat (mg/d)	44.9 (43.8–46.1)	21.6
Choline-grains and bakery (mg/d)	40.1 (39.3–40.8)	19.7
Choline-dairy products (mg/d)	39.4 (38.3–40.5)	19.6
Choline-fruits and vegetables (mg/d)	33.4 (32.6–34.1)	16.5
Choline-eggs (mg/d)	27.0 (26.1–28.0)	14.0
Choline-nuts (mg/d)	4.09 (3.93–4.25)	2.32
Choline-legumes (mg/d)	1.76 (1.69–1.82)	1.08
Choline-fats and oils (mg/d)	1.61 (1.55–1.67)	0.97
Betaine-grains and bakery (mg/d)	56.8 (55.6–58.2)	72.2
Betaine-fruits and vegetables (mg/d)	6.35 (6.16–6.55)	11.5
Betaine-meat (mg/d)	5.83 (5.68–5.60)	9.87
Betaine-dairy products (mg/d)	1.78 (1.72–1.83)	3.31
Betaine-nuts (mg/d)	0.59 (0.58–0.60)	0.88
Betaine-eggs (mg/d)	0.49 (0.44–0.55)	0.64
Betaine-legumes (mg/d)	0.064 (0.062–0.067)	0.13
Betaine-fats and oils (mg/d)	0.007 (0.006–0.008)	0.01

Data presented as geometric mean (95% prediction interval)

**Fig. 2:**
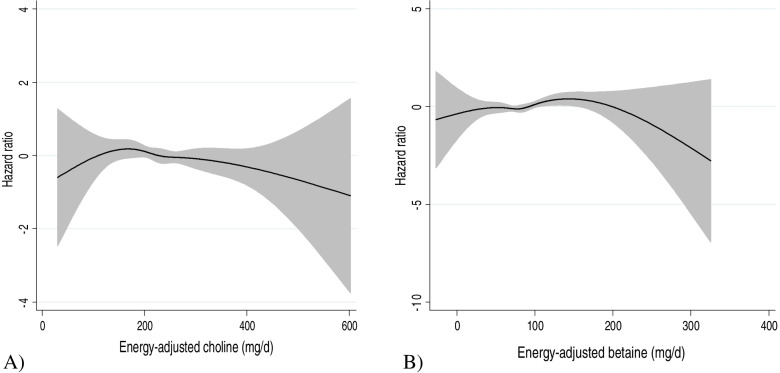
Restricted cubic spline model to assess the association between CVD and **A.** total choline and **B.** betaine

Over the follow-up periods, there were 187 incident CVD (166 incident CHD and 21 incident stroke). Results of Cox proportional hazard regression indicated no association between dietary energy-adjusted total choline (HR: 0.80; 95% CI: 0.56–1.23) nor energy-adjusted betaine (HR: 0.96; 95% CI: 0.64–1.44) and the incidence of CVD (Table 3). Furthermore, every 50 mg/d increase in energy-adjusted total choline (HR: 0.94; 95% CI: 0.83–1.07) and every 25 mg/d increase in energy-adjusted total betaine (HR: 0.95; 95% CI: 0.85–1.07) was not associated with an increased risk of CVD. The Schoenfeld residuals revealed no indication of PH assumption violation (*P* = 0.95). Restricted cubic spline curves did not show a significant nonlinear association between energy-adjusted choline and betaine and the risk of CVD (Fig. 2).

**Table 3:** Hazard ratios (95% CI) of CVD by total choline and betaine tertile

Dietary intake	Tertile 1	Tertile 2	Tertile 3	P trend	Continuous	P trend
**Total choline intake**						
Person years	9524	9418	9489		28,431	
No. of cases	63	69	55		187	
Median (mg/d)	176	228	289		Per 50 mg/d	
Age and sex-adjusted model	1	0.98 (0.70–1.36)	0.80 (0.56–1.14)	0.22	0.93 (0.84–1.04)	0.21
^a^Multivariate-adjusted model	1	0.97 (0.67–1.39)	0.80 (0.56–1.23)	0.31	0.94 (0.83–1.07)	0.39
**Betaine intake**						
Person years	9534	9497	9401		28,431	
No. of cases	57	56	74		187	
Median (mg/d)	56.4	80.7	110		Per 25 mg/d	
Age and sex-adjusted model	1	0.99 (0.69–1.41)	0.96 (0.68–1.35)	0.82	0.97 (0.88–1.07)	0.59
^b^Multivariate-adjusted model	1	1.07 (0.72–1.58)	0.96 (0.64–1.44)	0.83	0.95 (0.85–1.07)	0.45

Cox proportional hazards regression was conducted

^a^Adjusted for sex, age, smoking, BMI, total energy intake, FSG, TG/HDL, meat, fruit, vegetable, and oil

^b^Adjusted for sex, age, smoking, BMI, total energy intake, FSG, TG/HDL, SBP, fruit, oil, and fiber

In Table 4, the association between individual choline forms and CVD is shown. The results of the Cox proportional hazard model indicated that subjects in the third tertile of FC had a 36% lower risk of CVD than those in the first tertile of FC (HR: 0.64; 95% CI: 0.42–0.98). In the continuous model, each 10 mg/d increase in sphingomyelin reduced the risk of CVD in model 1 by 31% (HR: 0.69, 95% CI: 0.49 to 0.98); however, in the fully adjusted model, the observed association was lost. We also found no significant association between each 10 mg/d increase in other forms of choline and the risk of CVD.

**Table 4:** Hazard ratios (95% CI) of CVD events by individual choline forms tertile

Individual choline forms	Tertile 1	Tertile 2	Tertile 3	P trend	Continuous	P trend
**Free choline (FC)**						
Median (mg/d)	45.8	57.6	71.4		Per 10 mg/d	
Age and sex-adjusted model	1	0.79 (0.55–1.14)	0.82 (0.58–1.68)	0.34	0.96 (0.88–1.05)	0.44
^a^Multivariate-adjusted model	1	0.79 (0.54–1.16)	0.64 (0.42–0.98)	0.04	0.90 (0.80–1.01)	0.08
**Glycerophosphocholine (GPC)**						
Median (mg/d)	30.5	43.5	59.5		Per 10 mg/d	
Age and sex-adjusted model	1	0.92 (0.66–1.28)	0.73 (0.51–1.04)	0.08	0.94 (0.86–1.03)	0.22
^a^Multivariate-adjusted model	1	0.98 (0.69–1.37)	0.75 (0.52–1.07)	0.11	0.95 (0.87–1.04)	0.28
**Phosphocholine (PC)**						
Median (mg/d)	7.69	11.4	15.9		Per 10 mg/d	
Age and sex-adjusted model	1	0.85 (0.60–1.20)	0.78 (0.55–1.11)	0.19	0.93 (0.84–1.04)	0.21
^a^Multivariate-adjusted model	1	0.83 (0.57–1.21)	0.70 (0.46–1.06)	0.09	0.94 (0.83–1.07)	0.39
**Phosphatidylcholine (PtC)**						
Median (mg/d)	67.7	95.8	138		Per 10 mg/d	
Age and sex-adjusted model	1	1.05 (0.76–1.45)	0.84 (0.58–1.21)	0.33	0.98 (0.95–1.01)	0.35
^a^Multivariate-adjusted model	1	1.13 (0.79–1.61)	0.95 (0.60–1.50)	0.81	1.00 (0.95–1.05)	0.84
**Sphingomyelin (SM)**						
Median (mg/d)	6.90	9.88	14.0		Per 10 mg/d	
Age and sex-adjusted model	1	1.08 (0.78–1.48)	0.70 (0.48–1.01)	0.06	0.69 (0.49–0.98)	0.04
^a^Multivariate-adjusted model	1	1.19 (0.84–1.68)	0.72 (0.47–1.13)	0.16	0.76 (0.49–1.19)	0.24

Cox proportional hazards regression was conducted

^a^Adjusted for sex, age, smoking, BMI, total energy intake, FSG, TG/HDL, meat, fruit, vegetable, and oil

Results of sensitivity analysis with energy-adjusted total choline did not show an association with CVD were detected among healthy adults or high-risk patients. In addition, individual analyses for energy-adjusted betaine were not significant (Table 5).

**Table 5:** Sensitivity analysis for association between dietary choline and betaine intake the risk of CVD

Dietary intake	Tertile 1	Tertile 2	Tertile 3	P trend
**Healthy subjects (*n*** **= 839)**				
**Choline intake**				
Age and sex-adjusted model	1	0.65 (0.25–1.65)	0.52 (0.20–1.33)	0.17
^a^Multivariate-adjusted model	1	0.83 (0.28–2.45)	0.75 (0.21–2.66)	0.65
**Betaine intake**				
Age and sex-adjusted model	1	0.36 (0.11–1.13)	0.62 (0.26–1.45)	0.30
^b^Multivariate-adjusted model	1	0.36 (0.10–1.28)	0.51 (0.15–1.74)	0.33
**High-risk patients (*n*** **= 1767)**				
**Choline intake**				
Age and sex-adjusted model	1	1.09 (0.75–1.58)	0.95 (0.64–1.41)	0.79
^a^Multivariate-adjusted model	1	1.02 (0.68–1.54)	0.87 (0.54–1.39)	0.53
**Betaine intake**				
Age and sex-adjusted model	1	1.15 (0.77–1.72)	1.06 (0.72–1.55)	0.82
^b^Multivariate-adjusted model	1	1.27 (0.82–1.96)	1.07 (0.68–1.69)	0.85

Cox proportional hazards regression was conducted

^a^Adjusted for sex, age, smoking, BMI, total energy intake, FSG, TG/HDL, meat, fruit, vegetable, and oil

^b^Adjusted for sex, age, smoking, BMI, total energy intake, FSG, TG/HDL, SBP, fruit, oil, and fiber

Table 6 shows the association between energy-adjusted total choline and betaine content of each food category and the incidence of CVD. There was no significant association between each 10 mg/d increase in the choline and betaine content of each food category and the outcome of interest.

**Table 6:** Hazard ratios (95% CI) of CVD events each 10 mg/d increase in choline and betaine content of each food category

Food category	Total choline^a^	P trend	Betaine^b^	P trend
**Milk and dairy products**				
Age and sex-adjusted model	0.96 (0.91–1.01)	0.19	0.50 (0.16–1.50)	0.21
Multivariate-adjusted model	0.97 (0.92–1.02)	0.30	0.56 (0.19–1.65)	0.30
**Meat**				
Age and sex-adjusted model	0.98 (0.94–1.02)	0.32	0.95 (0.70–1.28)	0.75
Multivariate-adjusted model	0.99 (0.94–1.06)	0.98	1.06 (0.79–1.41)	0.69
**Eggs**				
Age and sex-adjusted model	0.98 (0.94–1.03)	0.55	0.55 (0.08–3.76)^c^	0.54
Multivariate-adjusted model	1.00 (0.95–1.05)	0.90	0.73 (0.11–4.84)^c^	0.74
**Grains and bakery products**				
Age and sex-adjusted model	0.99 (0.92–1.05)	0.76	0.99 (0.95–1.03)	0.67
Multivariate-adjusted model	0.98 (0.91–1.05)	0.66	0.98 (0.93–1.03)	0.52
**Fruit and vegetables**				
Age and sex-adjusted model	1.02 (0.96–1.09)	0.43	1.00 (0.87–1.15)	0.92
Multivariate-adjusted model	0.97 (0.81–1.16)	0.75	0.97 (0.82–1.14)	0.74
**Legumes**				
Age and sex-adjusted model	1.07 (0.70–1.63)	0.75	1.22 (0.53–2.80)	0.62
Multivariate-adjusted model	1.14 (0.75–1.73)	0.51	1.16 (0.51–2.63)	0.71
**Nuts**				
Age and sex-adjusted model	1.00 (0.82–1.21)	0.98	0.49 (0.04–5.42)^c^	0.56
Multivariate-adjusted model	0.99 (0.81–1.21)	0.94	0.58 (0.05–6.46)^c^	0.66
**Fats and oils**				
Age and sex-adjusted model	1.09 (0.51–2.31)	0.81	1.05 (0.008–145.25)^c^	0.98
Multivariate-adjusted model	1.69 (0.49–5.82)	0.40	2.02 (0.01–230.53)^c^	0.77

Cox proportional hazards regression was conducted

^a^Final model were adjusted for sex, age, smoking, BMI, total energy intake, FSG, TG/HDL, meat, fruit, vegetable, and oil

^b^Final model were adjusted for sex, age, smoking, BMI, total energy intake, FSG, TG/HDL, SBP, fruit, oil, and fiber

^c^HR (95% CI) was reported per 1 mg/d increase in intake of variable

## Discussion

In the present study, dietary energy-adjusted total choline and betaine intake were not associated with the risk of CVD in the general population. Among individual choline forms, there was an inverse association between FC intake and the risk of CVD. No relationship between other forms of choline and incidence of CVD was found. We also found no relationship between choline and betaine obtained from food categories and the outcome of interest. Results of sensitivity analysis indicated the association between energy-adjusted choline and betaine and CVD had the same pattern among healthy adults and high-risk patients.

Dietary choline and betaine were estimated by some previous studies. According to the eight European countries, the estimated choline intake ranged from 269 to 450 mg/d in adults [31]. Similar results were reported in the United States, whereby estimated intakes varied from 260 to 404 mg/d [32–34]. In comparison to prior findings, the observed values in our study were fairly low. However, our findings were comparable to those of Chinese studies, which found that estimated choline intakes ranged from 15865 to 297 mg/d [34–36]. In past studies conducted, dietary intakes of betaine were reported to range between 64.6–266 mg/d worldwide [14, 16, 19, 34, 36, 37]. All studies applied the USDA database to estimate choline and betaine intake. Nonetheless, due to variation in the number of FFQ items, intake of choline and betaine may not be similar between studies [36]. In addition, discrepancies in dietary assessment tools, adjusting for total energy, dietary pattern, and characteristics of the population, may be involved in different observed values among studies [38]. According to the National Academies of Medicine (NAM), the recommended adequate intake (AI) for choline is 550 and 425 mg/d for males and females, respectively [39]. The European Food Safety Authority (EFSA) established choline dietary reference values of 400 mg/d in 2016 [40]. However, no recommendations for Western Asian countries have been presented. It is important to note that in the present study, the mean intake of choline was below the AI values. In previous cohorts, most people did not meet the AI of choline [14, 16, 37, 41]. Notably, there is a lack of data about average requirements of choline, so further investigations to establish choline requirements are warranted.

This study was not the first to report that there is no significant link between dietary choline and betaine and CVD in the general population. Over an eight-year period, data from 16,165 CVD-free women in the PROSPECT-EPIC cohort revealed no significant relationship between dietary choline and betaine and the risk of total CVD, CHD, and cerebrovascular accident (CVA) [37]. Results of the Atherosclerosis Risk in Communities (ARIC) and Takayama studies also confirmed these findings [16, 42]. On the contrary, Mazidi et al. [41] in a prospective study (6.5-year of follow-up) reported that subjects in the highest quartile of choline had a 23% (HR: 1.23, 95% CI: 1.09 to 1.38) higher risk of all cause-mortality, 33% (HR: 1.33, 95% CI: 1.19 to 1.48) CVD mortality, and 30% (HR: 1.30, 95% CI: 1.02 to 1.66) stroke mortality than those in the first quartile. In the Jackson Heart Study, choline consumption is inversely associated with stroke incidents; while betaine indicated a positive association with CHD incidents [14]. While in the Takayama study, dietary intake of betaine was inversely linked to CHD mortality among Japanese males [42]. A meta-analysis of six studies involving 184,010 subjects, 18,076 CVD events, and 5343 CVD deaths found that neither choline (RR: 1.00, 95% CI: 0.98 to 1.02) nor betaine (RR: 0.99, 95% CI: 0.97 to 1.02) were related to CVD events [8].

Although all these studies had prospective designs, their conflicting results may be related to different sample sizes, various follow-up periods, and disparities in subjects’ geographical location, race, or residual confounders. Besides, inconsistencies in findings may be attributed to dietary patterns [8, 42]. In the present study, meat and dairy products supplied the highest amounts of choline, and grain was a good source of betaine. Another cohort has reported similar findings [16]. Eggs were the main source of choline in Nagata et al. [42] as well. But seafood was the main food source of betaine, which may explain an inverse association between betaine and mortality from CHD. Seafood is the major source of omega-3 polyunsaturated fatty acid (PUFA), which has been shown to protect against CVD in many studies [30, 43–47].

Furthermore, differences in the results of studies may be due to variations in gut microbiota composition across populations [48]. TMAO is formed by the gut microbiota metabolism of carnitine, choline, and betaine and is positively associated with CVD risk [11, 49]. Results of a new study indicated a significant relationship between nine gut bacterial species and TMAO [50]. Therefore, differences in the gut microbiota of subjects may have an impact on TMAO development [8]. The document revealed that some diseases, such as hypertension, obesity, and diabetes, alter gut microbiota composition [51, 52]. In the present study, we stratified subjects into two groups: healthy subjects and patients at high-risk of CVD who suffered from hypertension, obesity, and diabetes. Then we re-analyzed the data, but the results of the study remained unchanged. Previous studies did not evaluate the relationship between dietary choline and betaine and CVD among high-risk patients. Hence, further investigations to explore the possible effects of health conditions on the association between dietary choline and CVD events are warranted.

To the best of our knowledge, this is the first study to examine the association between individual choline forms and CVD . Unfortunately, there are just a few research reports on individual choline forms. Zheng et al. [53] showed a positive association between PtC and deaths from CVD among American adults. In contrast, Nagata et al. [42] did not find any association between PtC and CVD deaths in Japanese adults. The distribution of individual choline form intakes found in this cohort is consistent with the pattern reported in the Norwegian cohort [38]. In the current study, an inverse association between dietary FC and CVD but not the other forms of choline was detected. FC, a water-soluble form, is the second contributor to total choline. Plant-based foods are the most important sources of dietary FC [38]. Several cohort studies have found an inverse association between higher adherence to plant-based foods and a lower risk of CVD [54, 55]. Individual components of plant-based foods have also been linked to  a lower risk of CVD in previous research [56, 57]. It is possible that the FC content of plant foods may contribute to reducing the risk of CVD. Future studies on the association of dietary FC with the risk of CVD events are needed.

In addition, we evaluated the association between the choline content of each food category and the risk of CVD and found no significant association. As mentioned above, in our study, meat was the major food source of choline. In past studies, the link between the choline content of food categories and outcomes of interest was not investigated. Several studies have assessed the relationship between choline food sources and TMAO levels. Most of them indicated a positive association between fish and TMAO [58–60]. Significant associations between egg and meat consumption and TMAO levels were also reported by some studies [61–64] but not all [58, 59, 65, 66]. In a cross-sectional study of 3973 adults, Mei et al. [50] revealed that fish, red meat, and eggs were the most dietary factors that significantly increased TMAO production. However, they noted that the association between red meat and TMAO is due to TMA production from carnitine. Their findings were in agreement with the results of a clinical trial that showed the carnitine content of red meat increased production of TMAO but not choline [67]. This survey can explain the lack of association between the choline content of meat and eggs with CVD in the current study. However, it should be noted that TMAO has been associated with increased CVD risk, but that association does not necessarily mean causation.

There are several strengths and limitations. The follow-up period was extended. Outcomes were not self-reported and were collected based on medical records. The FFQ applied in our study was also validated to assess dietary intake of foods that are sources of choline and betaine. In addition, we assessed the association between individual choline forms and the choline and betaine content of each food category with outcomes of interest. However, the main limitation was that dietary intake of choline and betaine were collected at baseline and they might have changed over the follow-up. Due to small numbers of stroke events or CVD-related deaths, we were not able to evaluate the association between total choline and betaine with the risk of these events. The applied FFQ was not validated for choline and betaine and is also affected by recall bias. As a result, we were not able to determine how effectively it estimates actual choline intake. Another limitation was the estimation of choline and betaine using the USDA database. Because geographical location, temperature, and processing of food may impact the choline and betaine content of foods, the USDA database may not reliably reflect their intakes in our population. In addition, there is limited data for choline and betaine content in some foods in the USDA, which may cause an underestimation of total intake of choline and betaine. Furthermore, residual confounders cannot be ruled out even after adjusting for possible confounders in the studies.

## Conclusion

In conclusion, our results indicated no association between dietary energy-adjusted total choline and betaine intake and the risk of CVD. Among individual choline forms, there was an inverse association between FC intake and risk of CVD. No relationship between other forms of choline and incident CVD was found. We also found no relationship between choline and betaine obtained from food categories and the outcome of interest. Results of sensitivity analysis indicated the association between energy-adjusted choline and betaine and CVD had the same pattern among healthy adults and high-risk patients.

## Data Availability

Not applicable.

## Abbreviations

CHD: Coronary heart disease
CVA: Cerebrovascular accident
CVD: Cardiovascular disease
FSG: Fasting serum glucose
FC: Free choline
FFQ: Food frequency questionnaire
GPC: Glycerophosphocholine
IHD: Ischemic heart disease
MAQ: Modifiable activity questionnaire
PAD: Peripheral artery disease
PC: Phosphocholine
Ptc: Phosphatidylcholine
SD: Standard deviation
SM: Sphingomyelin
TLGS: Tehran Lipid and Glucose Study
TMAO: Trimethylamine N-oxide
USDA: United States Department of Agriculture
